# A chromosomal-level genome assembly of *Araneus marmoreus* Schenkel, 1953 (Araneae: Araneidae)

**DOI:** 10.1038/s41597-025-05215-1

**Published:** 2025-05-24

**Authors:** Lu-Yu Wang, Lin Xiao, Tian-Yu Ren, Ling-Xin Cheng, Jun-Han Xiong, Zheng Fan, Zhi-Sheng Zhang

**Affiliations:** Key Laboratory of Eco-environments in Three Gorges Reservoir Region (Ministry of Education), School of Life Sciences, Southwest University, Chongqing, 400715 China

**Keywords:** Genome, Zoology

## Abstract

The marbled orb-weaver spider, *Araneus marmoreus* (Araneae: Araneidae), is distinguished by its unique inflated, pumpkin-like abdomen. Numerous genome studies have been conducted on Araneidae species, providing insights into their unique biological traits. However, studies on *A. marmoreus* remain limited, despite its ecological significance and intriguing morphology. The lack of a high-quality reference genome has further hindered in-depth exploration of its evolutionary biology and ecological dynamics. Here, we present a chromosome-level genome assembly for *A. marmoreus*, generated using a combination of Illumina, PacBio, and Hi-C sequencing technologies. The assembled genome is 2.39 Gb in size, comprising 13 chromosomes, with a scaffold N50 of 181.8 Mb and a contig N50 of 721.3 kb. The assembly achieved a BUSCO completeness score of 97.1% (n = 2,934), including 91.0% complete and single-copy BUSCOs and 6.1% complete and duplicated BUSCOs. Repetitive sequences accounted for 59.25% of the genome, and 23,381 protein-coding genes were annotated. This high-quality genome provides a valuable resource for advancing research into the evolutionary genomics and ecological dynamics of *A. marmoreus*.

## Background & Summary

Spiders, as predatory arthropods, exhibit an extraordinary diversity, with more than 136 families and 52 thousand extant species described to date^1^. One of the largest spider family, Araneidae is particularly notable, known as orb-web weaving spiders, comprising more than 3.1 thousand species globally^1^ and considered one of the species-rich groups of spiders^2^. Most members of Araneidae heavily rely on their orb-web, a multifunctional tool for prey capture, communication, courtship, and mating^3–5^ and have been central to research on spider silk^6,7^, web-building behaviors^4^ and sexual size dimorphism^8^.

**Fig. 1:**
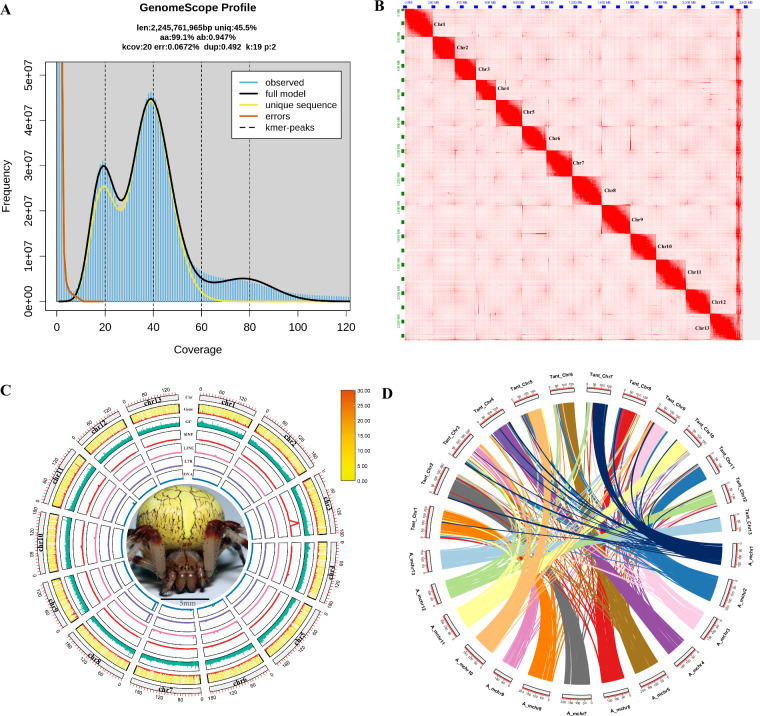
The genome analysis of orb-weaver spider *Araneus marmoreus*. (**A**) The survey analysis of *A. marmoreus*. (**B**) Heatmap of chromosome interactions in *A. marmoreus*. (**C**) Circos plot of distribution of the genomic elements in *A. marmoreus*. The inner ring contains a picture of orb-weaver spider. The outer rings of the circle represent means bellow, respectively: Chr: chromosomes, Gene: distribution of genes, GC: GC content; SINE: short interspersed nuclear element, LINE: long interspersed nuclear elements, LTR: long terminal repeat, DNA: DNA transposable elements. (**D**) Genomic synteny between *A. marmoreus* and *Trichonephila antipodiana*.

The marbled orb-weaver, *Araneus marmoreus* is characterized by the female’s inflated, pumpkin-like abdomen (Fig. 1). Adults display vibrant yellow to orange coloration, with black markings and banded legs. The species spins typical large orb-webs (>50 cm) between tall grasses, shrubs, and forest edges. Their life cycle is seasonal: adults mate in late summer, lay eggs, and perish after the breeding season^9,10^.

Up to now, although the genomes of 15 Araneidae spiders have been sequenced, this is insufficient for in-depth research on Araneidae spiders. The genus *Araneus*, in particular, lacks a chromosome-level genome. To fill this gap, we assembled a chromosome-level genome of *A. marmoreus* using PacBio HiFi, Illumina, and Hi-C sequencing technologies. The genome was annotated to identify repetitive elements, non-coding RNAs, and protein-coding genes. This high-quality genome provides a valuable foundation for further studies on Araneidae evolution and the genetic basis of orb-weaving spiders’ adaptations.

## Methods

### Sample collection and sequencing

The female specimens of *Araneus marmoreus* were collected from Chifeng City, Inner Mongolia Autonomous Region, China. The cephalothorax of the spiders, excluding the abdomen, was used for Illumina and PacBio sequencing, while leg muscle tissue was utilized for Illumina RNA-seq and Hi-C sequencing. The samples were first ground in liquid nitrogen, stored on dry ice, and subsequently sent to Berry Genomics (Beijing, China) for sequencing. Genomic DNA was extracted using the Qiagen Blood & Cell Culture DNA Mini Kit following the manufacturer’s protocol, optimized for PacBio and Illumina sequencing. PacBio sequencing employed Sequel II libraries with a 15 kb insert size, prepared using the SMRTbell™ Template Prep Kit 1.0-SPv3. Paired-end reads (150 bp) were generated using the Illumina NovaSeq platform for genome survey analysis and Hi-C sequencing. Total RNA was extracted from an adult female *A. marmoreus* using TRIzol (Invitrogen, USA) according to the manufacturer’s instructions and sequenced on the Illumina NovaSeq platform. We totally obtained 609.33 Gb clean data, including 149.68 Gb illumina reads (65×), 246.85 Gb Pacbio reads (107×), 204.81 Gb Hi-C reads (86×), and 7.99 Gb RNA reads (Table 1).

**Table 1:** The counts of the raw sequence data used for this study.

Pair-end	Clean data (Gb)	Sequencing coverage (×)	Insert sizes
Illumina reads	149.7	65	300 bp
PacBio reads	246.85	107	20 Kb
Hi-C	214.21	93	300 bp
RNA	7.99	—	300 bp
Total	637.4	—	—

### Survey analysis

First we used the “clumpify.sh” and “bbduk.sh” tools of BBTools suite v38.67^11^ to filter the Illumina reads. Then the filtered reads were feed to the “khist.sh” tool to estimate the k-mer distribution. And the software of GenomeScope v1.0.0^12^ was used to calculate genome size with the maximum k-mer coverage cutoff was set to 10,000 and the k-mer sets 19. For results, the estimated genome size of *A. marmoreus* was 2.24 Gb, the heterozygosity was 0.94% (Fig. 1A).

### Genome assembly

We used the softwere of Flye v2.5^13^ to assembly the draft assemble genome through the PacBio long reads with minimum overlap between reads (-m) set to 3000. Then the software of Purge Haplotigs v1.1.0^14^ was used to remove the heterozygous regions from the draft assembly genome. Next, the softwere of NextPolish v1.0.5^15^ was used to polished the assembly genome from last step with Illumina reads. And the software of Minimap2 v2.12^16^ was used to align the reads with the assembly. Finally, the software of Juicer v1.6.2^17^, 3D-DNA v.180922^18^, and Juicebox were used to obtain the chromosome-level assembly with Hi-C reads. In additation, we also remove the potential contaminants in the chromosome-level assembly through blast the NCBI nucleotide and UniVec databases with the software of HS-BLASTN^19^ and BLAST + (blastn) v2.7.1^20^. And the software of BUSCO v5.2.2^21^ pipeline was used to value the genome completeness with the arachnida_odb10 database (n = 2,934). In total, we obtained the chromosome assembly genome of *A. marmoreus* with the genome size of 2.39 Gb, scaffold N50 was 181.79 Mb, and contig N50 was 721.29 kb. A total of 13 chromosomes were assembled (Fig. 1B, Table 2), each larger than 100 Mb, and the Hi-C sequence was attached to the chromosome at a rate of 99.7%. The assembly achieved a BUSCO completeness score of 97.1% (n = 2,934), including 91.0% complete and single-copy BUSCOs and 6.1% complete and duplicated BUSCOs.

**Table 2:** Chromosome length information of *Araneus marmoreus*.

Chr_ID	Length(bp)	N_counts	GC_counts
A_mchr1	171,155,503	626,000	55,501,834
A_mchr2	205,971,813	689,500	66,073,220
A_mchr3	180,742,290	728,500	58,328,606
A_mchr4	181,895,792	636,000	58,214,867
A_mchr5	202,412,637	672,500	64,757,804
A_mchr6	181,788,316	570,000	58,234,538
A_mchr7	210,192,010	576,500	67,214,868
A_mchr8	201,540,963	656,500	64,121,461
A_mchr9	142,924,705	543,500	46,111,714
A_mchr10	183,047,159	673,000	59,032,794
A_mchr11	177,740,426	560,000	57,521,645
A_mchr12	147,842,533	131,000	47,176,265
A_mchr13	154,796,914	120,000	49,332,879

### RNA assembly

The clean RNA illumina reads were mapped to the assembly genome by the HISAT2 v 2.2.0^22^. Then using the Stringtie v2.1.3^23^ to assemble the transcripts.

### Genome annotation

Before genome annotation, the repetitive elements of the genome was first identified and softmasked by the software of RepeatModeler v2.0.1^24^ and RepeatMasker v.4.1.4^25^ through ab initio and homology-based searching with the Dfam database and RepBase RepeatMasker Edition database. In total, about 59.25% of assembly genome was annotated as repetitive elements, including 10.93% of DNA transposon elements, 3.21% of long terminal repeats (LTRs), 0.18% of long interspersed nuclear elements (LINEs), 0.18% short interspersed nuclear elements (SINEs), 41.48% of unclassified elements, 0.03% small RNAs, 0.01% satellites, 0.61% simple repeats, and 0.15% low-complexity regions (Table 3, Fig. 1C).

**Table 3:** Statistics of the repetitive sequences identified in *Araneus marmoreus*.

Type	Number	Length(bp)	% of genome
**Retroelements**	220,272	119,751,794	5.02%
**SINEs**:	28,715	4,270,438	0.18%
Penelope	6,497	3,574,939	0.15%
**LINEs**:	92,166	38,792,490	1.63%
L2/CR1/Rex	10,453	2,788,407	0.12%
R1/LOA/Jockey	46,242	21,794,713	0.91%
RTE/Bov-B	1,443	131,015	0.01%
L1/CIN4	3,433	182,977	0.01%
**LTR elements:**	99,391	76,688,866	3.21%
BEL/Pao	30,045	21,486,643	0.90%
Ty1/Copia	17,218	11,024,729	0.46%
Gypsy/DIRS1	46,852	43,181,302	1.81%
Retroviral	3,993	194,005	0.01%
**DNA transposons**	755,796	260,838,922	10.93%
hobo-Activator	241,546	70,189,393	2.94%
Tc1-IS630-Pogo	293,735	100,772,585	4.22%
MULE-MuDR	9,743	2,474,349	0.10%
PiggyBac	18,855	8,103,643	0.34%
Tourist/Harbinger	3,459	1,105,163	0.05%
Other (Mirage, P-element, Transib)	14,260	6,673,502	0.28%
**Rolling-circles**	51,678	16,764,704	0.70%
**Unclassified:**	4,145,591	997,688,542	41.81%
**Total interspersed repeats:**		1,378,279,258	57.75%
**Small RNA:**	10,787	731,810	0.03%
**Satellites:**	3,114	210,563	0.01%
**Simple repeats:**	322,829	14,655,640	0.61%

For gene structure annotation, we used the maker v3.01.04^26^ pipline based on ab-initio, EST and homologous proteins evidence. For ab-initio prediction, the software GeneMark-ETP v4.68_lic93 and Augustus v3.5.0^27^ were employed for initially trained using the BRAKER v3.0.2^28^. For EST evidence, the RNA transcripts were fed to maker pipline via the “est” option. For protein homology-based evidence, we downloaded the protein sequences of *Bombyx mori* (GCA_030269925.2), *Drosophila melanogaster* (GCA_000001215.4), *Parasteatoda tepidariorum* (GCA_000365465.3), *Stegodyphus mimosarum* (GCA_000611955.2) from NCBI, and *Trichonephila antipodiana* from GigaDB. And the proteins was fed to the maker pipline via the “protein” option. As the results, 23,381 protein-coding genes were identified, with an average length of 28,771.1 bp. Each gene exhibited an average of 6.91 exons, 6.75 CDS. The proteins annotated achieved a BUSCO completeness score of 97.8% (n = 2,934), including 85.8% complete and single-copy BUSCOs and 12% complete and duplicated BUSCOs.

For gene function annotation, the software EggNOG-mapper v2.1.10^29^, Diamond v2.0.14.152^30^, and InterProScan v5.48–83.0^31^ were used to identify gene ontology (GO), expression coherence (EC), Kyoto Encyclopedia of Genes and Genomes pathways (KEGG), KEGG orthologous groups (KOs), and clusters of orthologous groups (COG) through eggNOG v5.0^32^ based on the CDD^33^, Gene3D^34^, Panther^35^, Pfam^36^, and Superfamily^37^ databases. In total, 22,737 (97.25%) genes were identified with functional annotations. As a result, 16,690 genes were annotated with GO terms, and 13,891 genes were annotated at least one KEGG pathway (Table 4).

**Table 4:** Statistics of the *Araneus marmoreus* genome protein-coding gene annotation.

Characteristics	Number	Percent (%)
Protein-coding genes	23,381	100
genes with InterProScan annotations	19,723	84.39
genes with GO items from InterProScan annotations	12,449	53.24
genes with MetaCyc items from InterProScan annotations	12,997	55.58
genes with Reactome items from InterProScan annotations	16,441	70.31
genes matching Uniprot records	22,751	97.30
genes labelled as “Uncharacterized protein”	5,014	21.44
genes labelled as “unknown function”	630	2.69
genes with eggNOG annotations	20,201	86.39
genes with GO items from eggNOG annotations	13,895	59.42
genes with Enzyme Codes (EC) from eggNOG annotations	12,213	52.23
genes with KEGG ko terms from eggNOG annotations	13,385	57.24
genes with KEGG pathway terms from eggNOG annotations	13,891	59.41
genes with GO items (combining InterProScan and eggNOG results)	16,690	71.38

The software of Infernal v1.1.4^38^ and tRNAscan-SE v2.0.9^39^ were used to identified the Non-coding RNAs (ncRNAs) and transfer RNAs (tRNAs). The analysis revealed a total of 9,818 ncRNAs in the *A. marmoroide* genome, including 12,193 tRNAs, 2,997 ribosomal RNAs, 60 snoRNA, 47 microRNAs, 544 small nuclear RNAs, 35 ribozymes, and 330 other ncRNAs (Table S1).

## Data Records

The raw data used in the manuscript including Illumina, PacBio, Hi-C, transcriptome and the genome assembly and annotation of *Araneus marmoreus* have been deposited at the ScienceDB (https://cstr.cn/31253.11.sciencedb.19518)^40^, and NCBI database with project number of PRJNA774480, BioSample number of SAMN23402377, genome number of GCA_050042785.1 (https://www.ncbi.nlm.nih.gov/datasets/genome/GCA_050042785.1/)^41^, and SRA number of SRR32918500, SRR32918501, SRR32918502, and SRR32918503 (https://identifiers.org/ncbi/insdc.sra:SRP575255)^42^.

## Technical Validation

The mapping reads for DNA and RNA illumina reads to the assembly genome were 93.90% and 82.40%. And the mapping rates of Hi-C sequence to the chromosome was 99.7%. The assembly completeness of BUSCO was 97.1% (n = 2,934), and the annotated proteins completeness of BUSCO was 97.8% (n = 2,934). We checked the synteny block between *Araneus marmoreus* and *Trichonephila antipodiana* of Araneidae (Fig. 1D), which showed that the *A. marmoreus* genome has a good genome synteny relationship with *T. antipodiana*. And we did the consensus quality (QV) values analysis by the software Merqury^43^ for evaluation of the assembly genome quality based on the illumina data, and the value was 36.8084.

## Supplementary information

Table S1 Summary of non-coding RNA

## Data Availability

No specific script was used in this work. All commands and pipelines used in data processing were executed according to the manual and protocols of the corresponding bioinformatic software.
